# Effects of *Schizochytrium* and micro-minerals on immune, antioxidant, inflammatory and lipid-metabolism status of *Micropterus salmoides* fed high- and low-fishmeal diets

**DOI:** 10.1038/s41598-020-64286-9

**Published:** 2020-05-04

**Authors:** Habte-Michael Habte-Tsion, Gagan D. Kolimadu, Waldemar Rossi, Keith Filer, Vikas Kumar

**Affiliations:** 1 School of Aquaculture and Aquatic Sciences, College of Agriculture, Communities and the Environment, Kentucky State University, Frankfort, KY United States; 2 Aquaculture Research Institute, Department of Animal and Veterinary Science, University of Idaho, Moscow, ID United States; 3 Aquaculture Research Center, Alltech, Nicholasville, KY United States

**Keywords:** Immunogenetics, Transcriptomics, Fat metabolism

## Abstract

A 12-week factorial experiment was conducted to investigate the interactive effects of dietary algal meal (*Schizochytrium* sp., AM) and micro-minerals (MM, either organic [OM] or inorganic [IM]) on the immune and antioxidant status, and the expression of hepatic genes involved in the regulation of antioxidants, inflammatory cytokines, lipid metabolism, and organ growth of largemouth bass (LMB; *Micropterus salmoides*) fed high-and low-fishmeal (FM) diets. For this purpose, two sets of six iso-nitrogenous (42% crude protein) and iso-lipidic (12% lipid) diets, such as high (35%) and low (10%) FM diets were formulated. Within each FM level, AM was used to replace 50% or 100% of fish oil (FO), or without AM (FO control) and supplemented with either OM or IM (Fe, Zn, Mn, Cu, and Se). Diets were fed to juvenile LMB (initial weight, 25.87 ± 0.08 g) to near satiation twice daily. The results indicated that FO replacement by dietary AM did not change the levels of most biochemical (ALB, AMY, TP and GLOB), antioxidants (SOD, GPx and GSH), and immune (IgM and lysozyme) parameters in LMB, except ALP and CAT. MM affected only hepatic GSH, with lower values in fish fed the OM diets. FM influenced the levels of ALP, AMY, GLOB, IgM, and MDA (*P* < *0.05*). A three-way interactive effect (P = 0.016) was found on IgM only, with lower levels in fish fed diet 12 (low-FM, AM100, OM). Subsequently, the relative expressions of hepatic antioxidants (Cu/Zn-SOD and GPx-4), inflammatory cytokines (TNF-α and TGF-β1), lipid metabolism (FASN and CYP7A1), and organ growth (IGF-I) related genes were affected by the dietary treatments, with interactions being present in Cu/Zn-SOD, TNF-α, TGF-β1, FASN and IGF-I. Overall, dietary AM could be used as an alternative to FO in low-FM diets without compromising the health of LMB, especially when it is supplemented with MM.

## Introduction

Algae have been recognized as an alternative source of fatty acids (especially eicosapentaenoic acid [EPA], docosahexaenoic acid [DHA], and arachidonic acid [ARA]) for use in aquafeeds^1^. Micro-algae, such as *Schizochytrium* sp., and other thraustochytrids are recognized as prominent, sustainable, and alternative sources of oils rich in long-chain n-3 polyunsaturated fatty acid (n-3 LC-PUFA) since fisheries providing food-grade fishmeal (FM) and fish oil (FO) have already reached their limit of sustainability^2–4^. Individually or in combination, micro-minerals (MM) including iron (Fe), zinc (Zn), manganese (Mn), copper (Cu) and selenium (Se) are involved in diverse physiological functions such as facilitating cofactors of several enzymes, antioxidant defense, reduction of inflammation, protein synthesis, and so on^5–7^. Thus, it is generally recommended to supplement MM into plant-based fish feeds, due to the above essential roles of MM, and their low levels in plant feed ingredients.

In fish nutrition studies, many parameters related to biochemical, immune, antioxidants, and inflammatory cytokine status have been used as vital indicators of fish health^4,8–10^. Liver is one of the main organs involved in accumulation of fats, such as PUFAs, which are essential for membrane function in fish^11^. However, a large content of these lipids implies a high risk of oxidative stress, because they are the major targets for reactive oxygen species (ROS)^12,13^. To maintain the endogenous ROS at relatively low levels and to attenuate the damage related to the high reactivity of ROS, fish are equipped with a variety of enzymatic and non-enzymatic antioxidant scavenging systems^14^. In liver, lipid metabolism can directly induce body inflammatory responses^15^, including pro- and anti-inflammatory responses (e.g., tumor necrosis factor alpha, [TNF-α] and transforming growth factor-β1, [TGF-β1]). Simultaneously, organ growth is under endocrine control, specifically through the growth hormone (GH) - insulin-like growth factor (IGF) axis^16^.

Largemouth bass (LMB; *Micropterus salmoides*) is a freshwater carnivorous teleost native to North America, which has been cultured in the United States since the 1890s^17^. Recently, LMB production has increased in the United States. It has been introduced to China and is becoming one of the most commercially valuable fish there^18^. Due to its carnivorous feeding behavior, formulated feeds for LMB fundamentally rely on FM and FO as critical dietary components. The evaluation of alternative, sustainably produced ingredients for the optimization of cost-effective and nutritious feeds for LMB could improve the profitability of aquaculture operations and encourage the expansion of LMB production in the United States and beyond. There are few reports on the health/ immunonutrition studies for LMB^18–29^ and to the best of our knowledge, there is no report about the potential interactive effects of FM, algal meal (AM), and MM (organic minerals [OM] vs inorganic minerals [IM]) on the growth, health, and signaling molecules involved in antioxidant and inflammatory cytokines responses or lipid metabolism and organ growth of LMB. In an earlier study we conducted, the complete replacement of FO by dietary AM supplemented with MM did not negatively affect the growth and feed utilization of LMB, even at low FM levels (Kolimadu *et al*., unpublished). Therefore, we designed this study to investigate the interactive effects of dietary FM, AM and MM (OM vs IM) including Fe, Zn, Mn, Cu and Se, on the biochemical, immune, antioxidant and inflammatory cytokines status of juvenile LMB fed high- and low-FM diets. In this study, nutrigenomics was used to investigate the relationship between the nutrients (FM, AM and MM) and target genes involved in antioxidant and inflammatory responses and in lipid metabolism and organ growth.

## Materials and methods

### Diet formulation and preparation

Two sets of six practical diets were formulated to each contain 42% crude protein (CP), 12% lipid, and an estimated 14.64 kJ/g digestible energy (DE). The first set of diets contained 35% FM as the primary protein source (these are considered the high-FM diets) while the second set of diets contained 10% FM (these are considered the low-FM diets). Within each FM level, dietary *Schizochytrium* sp. meal (AM; Table 1) replaced 50% (AM50) or 100% (AM100) of dietary FO or without AM (AM0, used as FO control), in diets supplemented with Fe, Zn, Mn, Cu, and Se, either in OM or IM forms (Table 2).

**Table 1:** Nutritional profile of spray dried *Schizochytrium sp*^a^.

Chemical composition (as is)	Level	Chemical composition (as is)	Level
Moisture (%)	3.70	*……Fatty acids profile (%)*	
Crude fat (%)	50.00	Myristic Acid	3.86
Crude fiber (%)	0.90	Myristoleic Acid	1.60
Carbohydrates (%)	24.88	Pentadecanoic Acid	<0.10
Protein (%)	19.22	Palmitic Acid	54.69
***Major minerals (%)***		Palmitoleic Acid	<0.10
Total ash	3.67	Margaric Acid	0.63
Sodium	0.10	Margaroleic Acid	<0.10
Phosphorus	0.47	Stearic Acid	1.80
Sulfur	0.74	Vaccenic Acid	<0.10
Potassium	0.55	Oleic Acid	<0.10
Calcium	0.34	Elaidic Acid	<0.10
***Trace minerals (ppm)***		Linoleic Acid	<0.10
Iron	13.00	Linolelaidic Acid	<0.10
Copper	2.00	Alpha-Linolenic Acid	<0.10
Zinc	36.00	Gamma-Linolenic Acid	<0.10
Selenium	0.13	Nonadecanoic Acid	<0.10
***Glyceride profile (%)***		Arachidic Acid	0.28
Diglycerides	4.69	Eicosenoic Acid	<0.10
Glycerol	<1.00	Eicosadienoic Acid	<0.10
Monoglycerides	3.81	Eicosatrienoic Acid	<0.10
Triglycerides	85.8	Homo-gamma-Linolenic acid	<0.10
		Arachidonic Acid	<0.10
***Fatty acids profile (%)***		Eicosapentaenoic Acid	0.28
Caproic Acid	<0.10	Heneicosanoic Acid	<0.10
Heptatonic Acid	<0.10	Behenic Acid	<0.10
Caprylic Acid	<0.10	Erucic Acid	0.53
Nonanoic Acid	<0.10	Docosadienoic Acid	0.43
Capric Acid	<0.10	Docosapentaenoic Acid	<0.10
Undecanoic Acid	<0.10	Docosahexaenoic Acid	27.20
Lauric Acid	<0.10	Tricosanoic Acid	<0.10
Tridecanoic Acid	<0.10	Lignoceric Acid	<0.10

^a^Source: Alltech, Nicholasvile, KY, USA.

**Table 2:** Formulation and composition of the experimental diets.

	High FM Diets						Low FM Diets					
	D1	D2	D3	D4	D5	D6	D7	D8	D9	D10	D11	D12
	AM0 IM	AM0 OM	AM50 IM	AM50 OM	AM100 IM	AM100 OM	AM0 IM	AM0 OM	AM50 IM	AM50 OM	AM100 IM	AM100 OM
***Ingredients (%, dry basis)***												
Menhaden meal	**35.0**	**35.0**	**35.0**	**35.0**	**35.0**	**35.0**	**10.0**	**10.0**	**10.0**	**10.0**	**10.0**	**10.0**
Poultry by-product meal	**0.0**	**0.0**	**0.0**	**0.0**	**0.0**	**0.0**	**25.0**	**25.0**	**25.0**	**25.0**	**25.0**	**25.0**
Algae meal	**0.0**	**0.0**	**7.2**	**7.2**	**14.1**	**14.1**	**0.0**	**0.0**	**5.7**	**5.7**	**11.2**	**11.2**
Conventional SBM	25.0	25.0	25.0	25.0	25.0	25.0	25.0	25.0	25.0	25.0	25.0	25.0
Wheat gluten	3.4	3.4	1.7	1.7	0.0	0.0	3.5	3.5	2.1	2.1	0.7	0.7
Wheat flour	21.0	21.0	21.0	21.0	21.0	21.0	21.0	21.0	21.0	21.0	21.0	21.0
Carboxymethyl cellulose	1.5	1.5	1.5	1.5	1.5	1.5	1.5	1.5	1.5	1.5	1.5	1.5
Menhaden oil	**7.58**	**7.58**	**3.79**	**3.79**	**0.0**	**0.0**	**6.10**	**6.10**	**3.05**	**3.05**	**0.0**	**0.0**
Vitamin premix^a^	0.6	0.6	0.6	0.6	0.6	0.6	0.6	0.6	0.6	0.6	0.6	0.6
Stay C (35% Vit. C)	0.3	0.3	0.3	0.3	0.3	0.3	0.3	0.3	0.3	0.3	0.3	0.3
Choline chloride	0.2	0.2	0.2	0.2	0.2	0.2	0.2	0.2	0.2	0.2	0.2	0.2
Analog mineral premix	**0.0**	**2.03**	**0.0**	**2.03**	**0.0**	**2.03**	**0.0**	**2.03**	**0.0**	**2.03**	**0.0**	**2.03**
KSU-AN1 mineral premix^b^	**2.0**	**0.0**	**2.0**	**0.0**	**2.0**	**0.0**	**2.0**	**0.0**	**2.0**	**0.0**	**2.0**	**0.0**
Calcium phosphate dibasic	0.0	0.0	0.0	0.0	0.0	0.0	2.0	2.0	2.0	2.0	2.0	2.0
Lysine HCl	0.0	0.0	0.0	0.0	0.0	0.0	0.2	0.2	0.2	0.2	0.2	0.2
L-methionine	0.20	0.20	0.20	0.20	0.20	0.20	0.30	0.30	0.30	0.30	0.30	0.30
Aphacel	3.22	3.19	1.51	1.48	0.13	0.10	2.30	2.27	1.05	1.02	0.00	0.00
***Diet Composition (dry basis)***^***c***^												
Dry matter (%)	91.1	91.3	91.1	91.3	91.5	91.5	91.4	91.1	92.3	92.4	91.8	91.9
Crude protein (%)	45.0	44.7	44.0	44.2	42.0	43.4	44.2	44.4	44.2	44.3	41.7	42.0
Crude fat (%)	12.1	11.8	11.9	13.1	12.9	14.1	13.9	11.0	11.9	12.8	12.7	13.4
Crude fiber (%)	4.0	4.3	3.5	2.9	2.3	1.9	3.7	3.4	2.6	2.8	1.8	1.6
Ash (%)	12.3	12.1	12.2	13.4	13.4	14.6	14.2	11.2	12.3	13.3	13.0	13.8
***Major minerals (%)***												
Calcium	2.51	2.43	2.44	2.33	2.92	2.47	2.40	2.45	2.63	2.51	2.69	2.49
Phosphorus	1.73	1.74	1.71	1.68	1.91	1.75	1.79	1.87	1.92	1.93	1.94	1.83
***Micro-minerals (ppm)***												
Iron	298.7	279.4	273.4	275.0	277.7	246.0	328.2	336.8	339.1	308.3	298.4	293.7
Zinc	97.3	94.7	86.4	89.8	91.4	88.4	100.0	99.0	92.5	95.0	95.1	95.3
Manganese	42.3	29.6	37.6	28.6	35.6	27.3	37.2	28.6	35.2	27.1	30.1	22.0
Copper	13.0	14.4	11.8	13.2	11.0	12.2	15.3	14.4	11.7	13.2	9.4	11.8
Selenium	1.52	1.39	1.50	1.47	1.65	1.54	1.16	1.11	0.84	1.08	1.11	0.91

FM, fishmeal; D, diet; AM, algae meal; AM0, without AM/ Fish oil (FO) control; AM50, 50% AM; AM100, 100% AM; IM, inorganic mineral; OM, organic mineral. Analog mineral premix is IM or OM premix. Bioplex® was used for OM treatments (Alltech, Nicholasvile, KY, USA).

^a^Provides per kg of diet: retinyl acetate (vitamin A), 3000 IU; cholecalciferol (vitamin D), 2400 IU; all-rac-α-tocopheryl acetate (vitamin E), 60 IU; menadionesodium bisulfite (vitamin K), 1.2 mg; ascorbic acid monophosphate (49% ascorbic acid, vitamin C), 120 mg; cyanocobalamine (vitamin B12), 0.024 mg; d-biotin, 0.168 mg; choline chloride, 1200 mg; folic acid, 1.2 mg; niacin, 12 mg; d-calcium pantothenate, 26 mg; pyridoxine-HCl, 6 mg; riboflavin, 7.2 mg; thiamin-HCl, 1.2 mg.

^b^Provides the following macro-minerals (g/100 g of dry matter): calcium (0.16), phosphorus (0.19), magnesium (0.04), sodium (0.13), potassium (0.27), chloride (0.11), and sulfur (0.30); and micro-minerals (mg/100 g of dry matter): iron (4.05), aluminum (0.04), iodine (0.46), copper (0.51), manganese (0.92), cobalt (1.0), zinc (2.75), selenium (0.03), and chromium (0.10) (Modified from Moon and Gatlin^56^).

^c^Analyzed diet composition and data are mean value of three replicates.

Diet preparation was carried out as follows: solid ingredients were mixed for 30 min using a Hobart mixer (A200; Hobart, Troy, Ohio, USA) followed by the addition of FO and water until the appropriate consistency for pelleting was achieved. The resulting moist diets were passed through a pelletizer with a 3.2 mm die to form pellet strands and air-dried for 24 hours to a moisture content <10%. Diet strands were broken up and sieved using a standard testing sieve (1-mm opening mesh; Fisher Scientific, Pittsburg, PA, USA), sealed in vacuum-sealed plastic bags, and stored in a freezer (−20 °C) until used. A subsample of each diet was collected for proximate and test-mineral composition analyses (Table 2).

### Fish, experimental facility and fish rearing

The use of experimental fish was under scientific research protocols of Kentucky State University (KSU) and complied with all relevant local and/or international animal welfare laws, guidelines and policies^30^. In addition, this study was funded by the Alltech-Kentucky State University Alliance (Project No. 17-AAKS-11509), and all the experimental protocols were approved by the Alliance. Juvenile LMB were obtained from F&L Anderson Fish Farm, Lonoke, AR, USA, and transported to the aquatic animal nutrition laboratory (AANL), School of Aquaculture and Aquatic Sciences, KSU, Frankfort, KY, USA; and were acclimatized to the experimental conditions for three weeks. During the acclimatization period, fish were hand-fed to apparent satiation with a 45% CP and 15% crude fat commercial feed (EXTR 450; Rangen Inc., Buhl, ID, USA).

The experiment was conducted in a closed recirculating freshwater aquaculture system (RAS) at AANL. At the commencement of the feeding trial, fish were hand-graded and stocked in each of thirty-six 110-L glass tanks at a stocking density of 15 fish (mean individual weight = 25.87 ± 0.08 g)/tank. Three tanks were randomly assigned to each experimental diet. Fish in each tank were hand-fed with their respective diet to apparent satiation twice daily (at 08:00 and 16:00 hours) for 12 weeks. All water quality parameters were maintained within the acceptable ranges for LMB, including temperature (25.27 ± 0.5 °C), pH (7.56 ± 0.03), dissolved oxygen (6.19 ± 0.04 mg/l), total ammonia nitrogen (0.44 ± 0.02 mg/l), nitrite nitrogen (0.28 ± 0.01 mg/l) and salinity (1.61 ± 0.03ppt). A 12-h photoperiod was provided by artificial lighting controlled by a timer.

### Sample collection

At the end of the 12-week feeding trial, sampling was conducted after a 24-h fasting. Three representative fish from each tank (9 fish per treatment) were anaesthetized with 100 mg/l of tricaine methanesulfonate (MS-222). Blood samples were obtained from the caudal vein using heparinized syringes and transferred to 2.0 ml tubes. Blood samples were centrifuged at 3,000 × *g* at 4 °C for 10 min. The supernatant was removed and stored at −80 °C for plasma biochemistry and immune parameters assays. Liver samples of the anaesthetized fish were quickly removed and stored at −80 °C, such as 9 samples per treatment for hepatic peroxide content, and antioxidants enzymes activity and 6 samples per treatment for gene expression assays.

### Biochemical, immune and antioxidant parameters measurement

Plasma biochemical parameters were analyzed using a comprehensive diagnostic profile kit from VetScan Analyzer (Abraxis, Union City, CA, USA). The following parameters were analyzed: alkaline phosphatase (ALP), amylase (AMY), globulin (GLOB), albumin (ALB), and total protein (TP)^10^. Plasma immune parameters, such as lysozyme^31^ and immunoglobulin M (IgM)^32^, were measured using commercial kits following manufacturer’s protocols (BioVision, Milpitas, CA, USA). Hepatic peroxide (MDA) and antioxidants, such as superoxide dismutase (SOD), catalase (CAT), glutathione peroxidase (GPx) and glutathione (GSH), were analyzed by spectrophotometric measurements using commercial kits (BioVision, Milpitas, CA, USA), according to Kumar *et al*.^4^ and Habte-Tsion *et al*.^9^.

### RNA isolation, reverse transcription and real-time quantitative PCR assay

RNA isolation, reverse transcription and real-time quantitative PCR analysis were conducted according to previous studies^9,33^. Briefly, total RNA was isolated from LMB liver samples using a TRIzol^®^ reagent (Invitrogen™, Carlsbad, CA, USA). To avoid genomic contamination, the extracted RNA was treated with RNase-Free DNase (Takara, Dalian, China). The quality and quantity of the isolated RNA were assessed using a NanoDrop™ One^C^ spectrophotometer (Thermo Scientific™, Madison, WI, USA). Complementary DNA (cDNA) was synthesized using the PrimeScript^TM^ RT reagent kit, following the manufacturer’s instructions (Takara, Dalian, China). Specific primers for most target genes (growth, metabolism, and immune-cytokine- and antioxidant-related genes) were designed using online resources according to the partial cDNA sequences of the target genes for *Micropterus salmoides* transcriptome analysis, using the published sequences of LMB or adopted from published articles (Table 3). All primers for the target genes and housekeeping gene were synthesized by a commercial company (Life Technologies Corporation, Grand Island, NY, USA).

**Table 3:** Primer sequences for Real-time qPCR.

Target gene	Primer	Sequences 5′-3′	Length	TM (^o^C)	Amplicon size (bp)	Accession No./ Reference
IGF-I	F	GATCACGTGGCATTGTGGAC	20	59.6	95	DQ666526
	R	AGCAGGCTTGCTAGTCTTGG	20			
CYP7A1	F	CATCTGTCAAGGCATTCGGC	20	59.6	99	KT827791
	R	CCTCACCCTGCAAGGTCTTC	20			
FASN	F	ATCCCTCTTTGCCACTGTTG	20	57.5	121	Yu *et al*.^29^
	R	GAGGTGATGTTGCTCGCATA	20			
Cu/Zn-SOD	F	CCACAGAAACTTACGCGACA	20	58.5	100	FJ030929
	R	AAATAAACGGTCCCGGTGGT	20			
GPx-4	F	AGGTTTACGCATCCTTGCCT	20	59.7	92	AY309440
	R	TGTAGGAATGGGCAAACTGCT	21			
TNF-α	F	CTTCGTCTACAGCCAGGCATCG	22	63.0	161	Yu *et al*.^29^
	R	TTTGGCACACCGACCTCACC	20			
TGFβ1	F	GCTCAAAGAGAGCGAGGATG	20	59.0	118	Yu *et al*.^29^
	R	TCCTCTACCATTCGCAATCC	20			
β-actin	F	ATCGCCGCACTGGTTGTTGAC	21	60.0	336	Chen *et al*.^23^
	R	CCTGTTGGCTTTGGGGTTC	19			

IGF-I, insulin-like growth factor I; CYP7A1, Cholesterol 7-alpha-monooxygenase; FASN, fatty acid synthase; Cu/Zn-SOD, Cu/Zn-superoxide dismutase; GPx-4, glutathione peroxidase-4; TNF-α, tumor necrosis factor-α; TGF-β1, transforming growth factor β1.

Real-time qPCR was used to determine mRNA levels for the target genes and performed according to standard protocols of the manufacturers^33^. Briefly, cDNA (2.0 µL) was reacted with 10.0 µL SYBR Premix Ex Taq II (2X), 0.8 µL forward primer (10 µM), 0.8 µL reverse primer (10 µM), 0.4 µL ROX^TM^ reference dye or dye II (50X), and 6.0 µL RNase-free distilled water in a final reaction volume of 20 µL. The real-time PCR was carried out in a StepOnePlus^TM^ Real-Time PCR System (Applied Biosystems, Foster City, CA, USA). The thermocycling conditions for the target genes were initiated with the denaturation step at 95 °C for 30 s followed by forty cycles of 95 °C for 5 s, 60 °C for 34 s, and 95 °C for the 30 s, 95 °C for 3 s, and 60 °C for 30 s. A melting curve analysis was performed (over a range of 50–95 °C) to verify that a single PCR product was produced. Threshold cycle number (C_T_) for each sample was determined using Applied Biosystems software and was related to the concentration of the target genes. The housekeeping gene for *M. salmoides*^23^ (β-actin) was used to normalize the expression levels of the target genes. After verifying that the primers were amplified with 100% efficiency, the expression results were analyzed using the 2^−ΔΔCt^ method^34^.

### Statistical analysis

All data were validated for normality and homogeneity of variances by Shapiro-Wilk and Levene’s tests, respectively. A three-way analysis of variance (ANOVA) was performed to detect the interactive effects of dietary FM (high and low), AM (control, and 50% and 100% FO replacements), and MM source (OM and IM). Statistical significance was considered as P < 0.05. If significant interactions were identified, the Tukey’s honest significant difference (HSD) test was used for mean separation. In the absence of significant interactions (additive model), the means of any significant factor with more than two levels were separated using Tukey HSD test. All statistical analyses were performed using Statistical Analysis System software (SAS; SAS Institute Inc., Cary, NC, USA). Results are expressed as mean ± standard error (SE).

## Results

### Plasma biochemical and immune parameters

Plasma biochemical (ALP, AMY, GLOB, ALB and TP) and immune (IgM and lysozyme) parameters are presented in Table 4. LMB fed low-FM diets displayed significantly higher ALP activity (P = 0.001), while the reverse was found for AMY activity (P = 0.009) and GLOB concentration (P = 0.020). Replacement of dietary FO by AM influenced the activity of ALP only, with its lower level in fish fed AM100 (P < 0.05), but not significantly different from the FO control (AM0). LMB fed diets 5 (high-FM, AM100, IM) and 6 (high-FM, AM100, OM) had lower ALP activities compared to fish fed diet 8 (low-FM, OM) while fish fed diet 6 also had lower plasma ALP activity than those fed diet 3 (high-FM, AM50, IM) (P = 0.004). A two-way interaction between dietary FM and MM was observed for ALP activity and GLOB concentration. There were no three-way interactive effects of FM, AM, and MM on the plasma biochemical parameters.

**Table 4:** Plasma biochemical and immune parameters of LMB fed the experimental diets for 12 weeks^a^.

				ALP (U/L)	AMY (mg/dL)	GLOB^++^ (mg/dL)	ALB (g/dL)	TP (g/dL)	IgM (µg/mL)	Lysozyme (mU/mL)
*Treatment means*										
Diet	FM level	AM level (%)	MM source							
D1	H	0	I	61.7 ± 1.7^abc^	164.3 ± 6.2	2.0 ± 0.1	2.2 ± 0.1	4.3 ± 0.1	997.7 ± 35.8^ab^	27.7 ± 1.0
D2	H	0	O	59.7 ± 1.5^abc^	174.0 ± 10.8	2.2 ± 0.1	2.1 ± 0.1	4.3 ± 0.2	984.7 ± 23.0^ab^	29.1 ± 2.2
D3	H	50	I	78.3 ± 11.0^ab^	134.3 ± 42.2	2.0 ± 0.2	2.2 ± 0.1	4.2 ± 0.2	963.4 ± 52.6^ab^	27.5 ± 1.1
D4	H	50	O	57.0 ± 4.6^abc^	166.0 ± 5.5	2.1 ± 0.0	2.2 ± 0.1	4.3 ± 0.2	1023.2 ± 39.8^ab^	32.1 ± 3.2
D5	H	100	I	52.7 ± 4.1^bc^	152.7 ± 2.0	2.1 ± 0.0	2.3 ± 0.1	4.4 ± 0.1	915.7 ± 16.0^ab^	28.6 ± 1.6
D6	H	100	O	44.0 ± 6.4^c^	180.0 ± 20.1	2.2 ± 0.1	2.1 ± 0.2	4.2 ± 0.2	1124.9 ± 144.8^a^	31.4 ± 4.3
D7	L	0	I	63.3 ± 12.3^abc^	126.3 ± 3.3	2.0 ± 0.1	2.1 ± 0.1	4.1 ± 0.1	872.2 ± 29.3^ab^	27.7 ± 1.0
D8	L	0	O	87.0 ± 7.9^a^	97.0 ± 28.5	1.8 ± 0.1	2.2 ± 0.2	4.0 ± 0.2	1034.1 ± 32.2^ab^	30.0 ± 1.7
D9	L	50	I	71.7 ± 2.7^abc^	131.0 ± 6.1	2.1 ± 0.1	2.2 ± 0.0	4.3 ± 0.2	912.3 ± 31.6^ab^	27.9 ± 1.7
D10	L	50	O	77.0 ± 8.5^abc^	104.3 ± 42.0	1.9 ± 0.1	2.4 ± 0.2	4.3 ± 0.3	929.1 ± 96.5^ab^	27.7 ± 0.9
D11	L	100	I	64.0 ± 3.2^abc^	157.0 ± 28.9	2.0 ± 0.1	2.4 ± 0.2	4.5 ± 0.1	968.0 ± 29.2^ab^	25.4 ± 1.0
D12	L	100	O	75.3 ± 2.3^abc^	123.7 ± 27.5	2.0 ± 0.1	2.3 ± 0.0	4.3 ± 0.1	816.5 ± 28.0^b^	26.3 ± 0.6
*Main effect means*										
FM level H L AM level (%) 0 50 100 MM source I O				58.9 ± 3.2	161.9 ± 7.7	2.1 ± 0.0	2.2 ± 0.0	4.3 ± 0.1	1001.6 ± 31.0	29.4 ± 1.0
				73.1 ± 3.1	123.2 ± 10.2	2.0 ± 0.0	2.3 ± 0.1	4.2 ± 0.1	922.0 ± 21.6	27.4 ± 0.6
				67.9 ± 4.6^ab^	140.4 ± 11.4	2.0 ± 0.1	2.2 ± 0.1	4.2 ± 0.1	972.2 ± 28.2	28.7 ± 0.8
				71.0 ± 4.1^a^	133.9 ± 14.4	2.0 ± 0.1	2.3 ± 0.1	4.3 ± 0.1	957.0 ± 29.6	28.2 ± 1.0
				59.0 ± 4.0^b^	153.3 ± 11.3	2.1 ± 0.0	2.3 ± 0.1	4.4 ± 0.0	956.3 ± 42.2	28.3 ± 1.2
				65.3 ± 3.1	144.3 ± 8.1	2.0 ± 0.0	2.3 ± 0.0	4.3 ± 0.1	938.2 ± 14.7	28.0 ± 0.5
				66.7 ± 4.0	140.8 ± 12.0	2.0 ± 0.1	2.2 ± 0.1	4.2 ± 0.1	985.4 ± 35.6	28.8 ± 1.1
*ANOVA (Pr* > *F)*										
FM level AM level MM source FM level × AM level FM level × MM source AM level × MM source FM level × AM level × MM source				0.001	0.009	0.020	0.172	0.649	0.035	0.094
				0.042	0.501	0.604	0.256	0.349	0.925	0.930
				0.716	0.801	0.922	0.683	0.649	0.206	0.313
				0.302	0.611	0.346	0.545	0.331	0.608	0.446
				0.004	0.064	0.020	0.227	0.733	0.306	0.083
				0.148	0.933	0.854	0.331	0.525	0.868	0.735
				0.927	0.938	0.760	0.940	0.971	0.016	0.673

FM, Fishmeal; H, high; L, low; AM, algal meal; MM, micro-mineral; I, inorganic; O, organic; ALP, alkaline phosphatase; AMY, amylase; GLOB, globulin; ALB, albumin; TP, total protein; IgM, immunoglobulin M.

^a^Data are mean value ± SE. We performed nine analysis per treatment; n = 9. Mean values in the same column with different superscript are significantly different (P < 0.05). ^++^= difference undetected by Tukey HSD.

A three-way interaction (P = 0.016) of FM, AM and MM was detected for IgM, with its higher concentration in the plasma of LMB fed diet 6 (high-FM, AM100, OM) compared to those fed diet 12 (low-FM, AM100, OM), while no significant differences were found among other treatments. Plasma lysozyme was not significantly affected by dietary treatments.

### Hepatic peroxide and antioxidants

Hepatic peroxide and antioxidant parameters including MDA, SOD, GPx and GSH, were not significantly affected by replacement of dietary FO with AM and no interactions were found (Table 5). Significantly higher MDA content was observed in the liver of LMB fed high-FM diets compared to those fed low-FM diets. LMB fed AM0 diets displayed lower (P = 0.002) CAT activity relative to those fed AM50 and AM100 diets. A slightly but significant effect of dietary MM was observed for GSH activity in the liver of LMB, which was higher in groups fed IM than those fed OM (P = 0.042).

**Table 5:** Hepatic peroxide and antioxidants of LMB fed the experimental diets for 12 weeks^a^.

				MDA(nmol/mL)	SOD (U/mL)	CAT (mU/mL)	GPx (U/mg)	GSH (µg/mL)
*Treatment means*								
Diet	FM level	AM level (%)	MM source					
D1	H	0	I	2.13 ± 0.41	1.29 ± 0.08	0.51 ± 0.26	1.43 ± 0.04	1.38 ± 0.02
D2	H	0	O	1.92 ± 0.35	1.46 ± 0.15	0.73 ± 0.15	1.49 ± 0.11	1.34 ± 0.00
D3	H	50	I	2.75 ± 0.66	1.48 ± 0.25	0.98 ± 0.40	1.37 ± 0.10	1.37 ± 0.01
D4	H	50	O	2.47 ± 0.43	1.29 ± 0.04	1.30 ± 0.27	1.55 ± 0.03	1.34 ± 0.01
D5	H	100	I	2.18 ± 0.47	1.39 ± 0.09	1.12 ± 0.19	1.50 ± 0.02	1.39 ± 0.04
D6	H	100	O	2.15 ± 0.33	1.17 ± 0.11	1.13 ± 0.20	1.45 ± 0.02	1.35 ± 0.01
D7	L	0	I	1.83 ± 0.30	1.40 ± 0.05	0.39 ± 0.11	1.42 ± 0.03	1.36 ± 0.01
D8	L	0	O	2.01 ± 0.32	1.35 ± 0.06	0.63 ± 0.16	1.46 ± 0.03	1.38 ± 0.01
D9	L	50	I	1.45 ± 0.33	1.49 ± 0.09	0.55 ± 0.13	1.51 ± 0.06	1.39 ± 0.03
D10	L	50	O	1.58 ± 0.22	1.39 ± 0.06	0.85 ± 0.42	1.43 ± 0.05	1.35 ± 0.01
D11	L	100	I	1.35 ± 0.29	1.33 ± 0.04	1.01 ± 0.19	1.47 ± 0.07	1.36 ± 0.02
D12	L	100	O	2.23 ± 0.27	1.37 ± 0.05	0.97 ± 0.19	1.52 ± 0.04	1.37 ± 0.01
*Main effect means*								
FM level H L AM level (%) 0 50 100 MM source I O				2.27 ± 0.18	1.35 ± 0.06	0.98 ± 0.11	1.47 ± 0.03	1.36 ± 0.01
				1.74 ± 0.12	1.39 ± 0.02	0.76 ± 0.09	1.47 ± 0.02	1.37 ± 0.01
				1.97 ± 0.16	1.38 ± 0.05	0.56 ± 0.10^b^	1.45 ± 0.03	1.36 ± 0.01
				2.06 ± 0.24	1.41 ± 0.07	0.92 ± 0.17^a^	1.46 ± 0.03	1.36 ± 0.01
				1.98 ± 0.18	1.31 ± 0.04	1.05 ± 0.09^a^	1.49 ± 0.02	1.37 ± 0.01
				1.95 ± 0.18	1.39 ± 0.05	0.80 ± 0.11	1.45 ± 0.03	1.37 ± 0.01
				2.06 ± 0.13	1.34 ± 0.04	0.95 ± 0.11	1.48 ± 0.02	1.35 ± 0.00
*ANOVA (Pr* > *F)*								
FM level AM level MM source FM level × AM level FM level × MM source AM level × MM source FM level × AM level × MM source				0.020	0.475	0.338	0.993	0.367
				0.934	0.416	0.002	0.674	0.926
				0.624	0.349	0.742	0.350	0.042
				0.176	0.887	0.337	0.879	0.652
				0.205	0.722	0.465	0.359	0.114
				0.600	0.399	0.806	0.768	0.694
				0.861	0.285	0.849	0.097	0.338

MDA, malondialdehyde; SOD, superoxide dismutase; CAT, catalase; GPx, glutathione peroxidase; GSH, glutathione.

^a^Data are mean value ± SE. We performed nine analysis per treatment; n = 9. Mean values in the same column with different superscript are significantly different (P < 0.05).

### Relative expression of hepatic antioxidants genes

**Figure 1:**
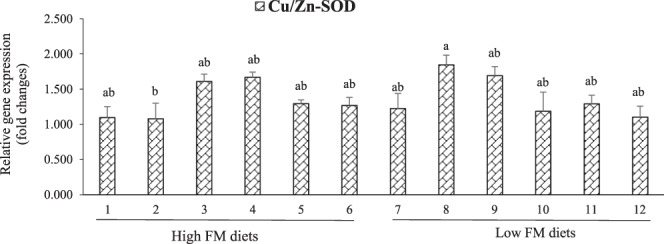
Interactive effects (P = 0.030) of dietary fishmeal (FM), algae meal (AM), and micro-mineral (MM) on the relative gene expression of hepatic copper/zinc-superoxide dismutase (Cu/Zn-SOD) in LMB fed the experimental diets for 12 weeks. Values are means with standard errors represented by vertical bars (n = 6). Mean values with different letters are significantly different (P < 0.05).

**Figure 2:**
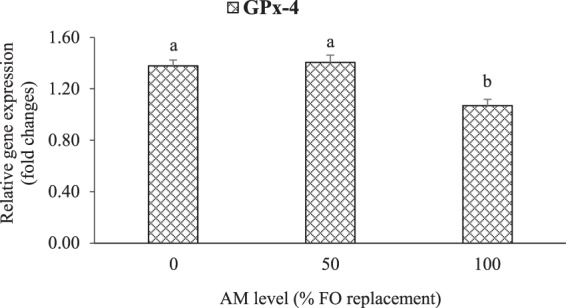
Effect (P < 0.001) of dietary AM (0, 50, and 100% FO replacements) on the relative gene expression of hepatic glutathione peroxidase 4 (GPx-4) in LMB fed the experimental diets for 12 weeks. Values are means with standard errors represented by vertical bars (n = 6). Mean values with different letters are significantly different (P < 0.05).

The relative expressions of antioxidants genes in LMB fed the experimental diets are shown in Figs. 1 and 2. A three-way interactive effect (P = 0.030) of dietary FM, AM, and MM was found on the relative gene expression of Cu/Zn-SOD, which was significantly down-regulated in fish fed diet 2 (high-FM, AM50, OM) compared to those fed diet 8 (low-FM, AM0, OM) (Fig. 1). The gene expression of hepatic glutathione peroxidase 4 (GPx-4) was affected (P < 0.001) by dietary AM only, with the lowest level observed in LMB fed AM100 (Fig. 2).

### Relative expression of hepatic pro- and anti-inflammatory cytokines genes

**Figure 3:**
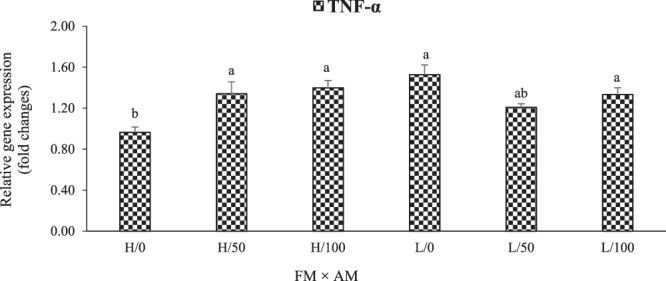
Interactive effects (P < 0.001) of dietary FM [high (H) and low (L)] and AM (0, 50, and 100% FO replacements) on the relative gene expression of hepatic tumor necrosis factor alpha (TNF-α) in LMB fed the experimental diets for 12 weeks. Values are means with standard errors represented by vertical bars (n = 6). Mean values with different letters are significantly different (P < 0.05).

**Figure 4:**
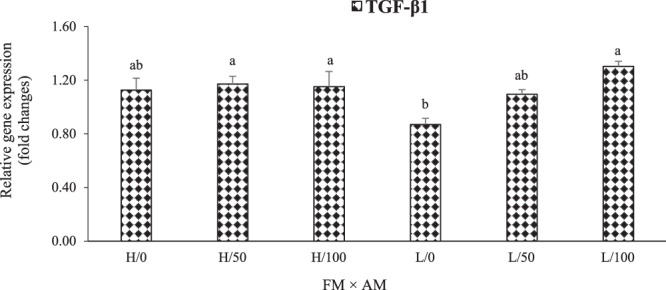
Interactive effects (P = 0.011) of dietary FM (high [H] and low [L]) and AM (0, 50, and 100% FO replacements) on the relative gene expression of hepatic transforming growth factor-β1 (TGF-β1) in LMB fed the experimental diets for 12 weeks. Values are means with standard errors represented by vertical bars (n = 6). Mean values with different letters are significantly different (P < 0.05).

Figures 3 and 4 presented the relative expressions of hepatic pro- and anti-inflammatory cytokines genes in LMB fed the experimental diets. Two–way interactive effects of dietary FM and AM were obtained on the relative gene expression of hepatic tumor necrosis factor alpha (TNF-α) (P < 0.001) and transforming growth factor-β1 (TGF-β1) (P = 0.011). In contrast to the other treatments, fish fed high-FM and AM0 diets had significantly lower TNF-α expression except compared those fed low-FM and AM50 diets (Fig. 3). Low-FM with AM0 level down-regulated the TGF-β1 expression compared to high-FM (with AM50, and AM100) and low-FM with AM100 treatments (Fig. 4).

### Relative expression of genes related to hepatic lipid metabolism and organ-growth

**Figure 5:**
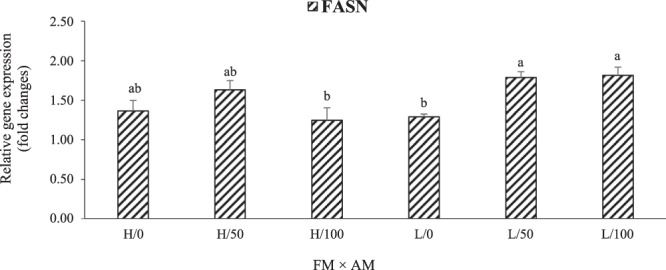
Interactive effects (P = 0.0173) of dietary fish meal (high [H] and low [L]) and AM (0, 50, and 100% FO replacements) on the relative gene expression of hepatic fatty acid synthase (FASN) in LMB fed the experimental diets for 12 weeks. Values are means with standard errors represented by vertical bars (n = 6). Mean values with different letters are significantly different (P < 0.05).

**Figure 6:**
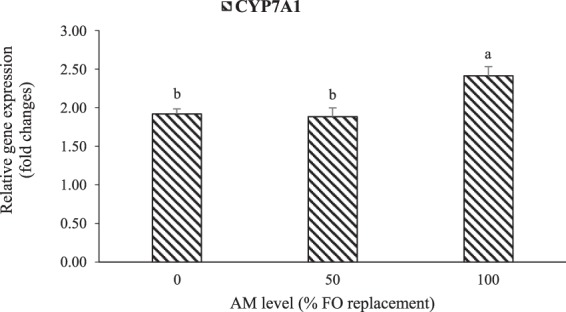
Effect (P < 0.001) of dietary AM (0, 50, and 100% FO replacements) on the relative gene expression of hepatic cholesterol 7-alpha-monooxygenase (CYP7A1) in LMB fed the experimental diets for 12 weeks. Values are means with standard errors represented by vertical bars (n = 6). Mean values with different letters are significantly different (P < 0.05).

The relative expressions of genes related to hepatic lipid metabolism are shown in Figs. 5 and 6. Interactive effects (P = 0.017) of dietary FM and AM were detected on the relative gene expression of hepatic fatty acid synthase (FASN) in LMB fed the experimental diets (Fig. 5). Dietary AM up-regulated the expression of FASN in fish fed the low-FM supplemented with AM50 and AM100 diets relative to those in low-FM with AM0 groups. AM was the only factor that significantly affected the expression of cholesterol 7-alpha-monooxygenase (CYP7A1) in LMB, which was significantly up-regulated in the groups fed AM100 in contrast to those fed AM0 and AM50 (Fig. 6).

**Figure 7:**
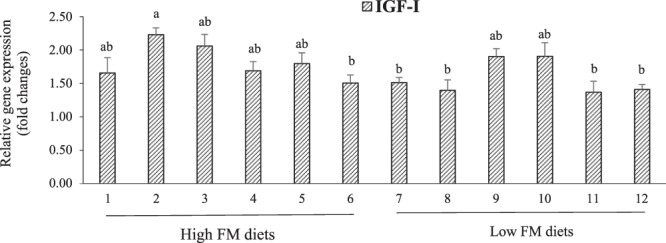
Interactive effects (P = 0.022) of dietary FM, AM, and MM on the relative gene expression of hepatic insulin-like growth factor I (IGF-I) in LMB fed the experimental diets for 12 weeks. Values are means with standard errors represented by vertical bars (n = 6). Mean values with different letters are significantly different (P < 0.05).

Figure 7 presented the relative expression of insulin-like growth factor I (IGF-I), a gene related to hepatic-growth in LMB fed the experimental diets. In general, LMB fed high-FM diets displayed greater expression of IGF-I than those fed low-FM diets (P = 0.007), while significantly higher expression of IGF-I was also found in LMB fed the low-FM supplemented with AM50 diets relative to low-FM with AM100 fed groups. These responses were not consistent across all treatments and a three-way interaction was observed (P = 0.022). The relative expression of IGF-I was significantly up-regulated in the group fed diet 2 (high-FM, AM0, OM) compared to those fed diets 6 (high-FM, AM100, OM), 7 (low-FM, AM0, IM), 8 (low-FM, AM0, OM), 11 (low-FM, AM100, IM), and 12 (low-FM, AM100, OM), while not significantly different from the remaining treatments (Fig. 7).

## Discussion

In aquatic animals, blood parameters are considered as important indicators for physiological conditions and health status in response to dietary treatments^8,9,35^. In this study, we did not find any significant three-way interactive effects of FM, AM, and MM on any analyzed plasma biochemistry parameters in LMB. Simultaneously, the replacement of dietary FO by AM did not influence most of the plasma biochemistry in LMB, except ALP. Similarly, no significant effects of dietary *Schizochytrium* sp., supplemented with either inorganic or organic MM (OM: Zn, Cu, Mn, Fe, and Se) were observed on blood plasma chemistry of Atlantic salmon^7^. Our recently published study revealed that dietary *Schizochytrium* meal could replace dietary FO up to 75% and play an imperative role as a source of essential fatty acids in shrimp (*Litopenaeus vannamei*) diets without compromising growth and health^4^. However, in the present study, there were significant differences in blood plasma ALP levels between treatments with 50% and 100% dietary FO replacements by AM, with ALP levels higher in fish fed AM50, but these were not significantly different compared to the AM0 treatments. Nonetheless, the highest level of plasma ALP in our study (87.0 U/L) is below the normal ranges that reported in Atlantic salmon (647–988 U/L)^36^. In humans, the level of ALP in a healthy adult range from 20–140 U/L; however, children tend to have significantly higher levels of ALP than adults because their bones are still growing^37^. The presence of ALP activity in plasma is directly related to the release of ALP enzymes from cells to the extracellular fluids and elevated ALP activity may occur when there is cell growth, tissue necrosis, or leakage of preformed ALP^38,39^. Therefore, in this study, the higher levels of plasma ALP in LMB fed AM50 could be related to the cell/ bone growth of juvenile LMB. Nevertheless, further study is needed to understand the specific mechanisms of the effects of dietary AM on plasma ALP in fish.

In teleost fish, producing immunoglobulins is a specific immune response after being stimulated by antigen and the IgM class is the predominant immunoglobulin in most fish species^40^. Lysozyme is a ubiquitous bacteriolytic enzyme that is part of the nonspecific defense mechanisms in most animals^41^. In this study, FO replacement by dietary AM did not affect the levels of the immune parameters such as IgM and lysozyme, in the plasma of LMB. However, LMB fed diet 6 (high-FM, AM100, OM) displayed significantly higher plasma IgM in contrast to those fed diet 12 (low-FM, AM100, OM), but not among other treatments. We also found interactive effects of dietary FM, AM and MM on the IgM content in LMB. Our findings suggest that dietary AM could maintain immune defense system/ health of LMB, even when replacing 100% of dietary FO, especially when supplemented with MM. This is in agreement with a study on Atlantic salmon that reported whole *Schizochytrium* sp.-based microalgae can provide a good alternative to the depleting marine resources of n-3 LC-PUFA without detrimental health effects^7,42^, even more pronounced for salmon fed OM^7^. Nevertheless, this is the first study to investigate the interactive effects of three factors (FM, AM and MM) in fish, therefore further study is needed.

Fish hepatic tissue has large quantities of PUFAs^11^, which implies a high risk of oxidative stress since these lipids are major targets for ROS^12,13^. The antioxidant defense system helps fish to maintain endogenous ROS at relatively low levels and to attenuate the oxidative damage induced by the high reactivity of ROS^14^. An increase in free radicals causes overproduction of MDA, which is one of the final products of lipid peroxidation in the cells. Thus, the MDA level is commonly known as a marker of oxidative stress^43^. Nogueira *et al*.^44^ reported that a decrease in MDA content is associated with an increase in antioxidant enzymes in the defense system, which indicates MDA is a toxic substance that is caused by free radicals. The key antioxidants in the antioxidant defense system include SOD, GPx, CAT, and GSH^13^. In fish, antioxidant enzymes activities correlate with nutritional factors^9,45^. In the present study, we did not find significant differences between the control (FO control) and AM treatments in the hepatic MDA, SOD, GPx and GSH levels of LMB, except higher CAT activity in LMB fed AM-diets. The significantly higher MDA content observed in the liver of LMB fed high-FM compared to low-FM diets might be related to the MDA content of dietary FM, which was not quantified in this study. Whereas IM supplementation supported significantly higher GSH in comparison to OM (1.37 ± 0.01 vs 1.35 ± 0.00), such narrow differences suggest limited practical significance.

The activities of antioxidant enzymes in fish are closely related to their corresponding gene expression levels^46^. In this study, there were no significant differences in the level of hepatic Cu/Zn-SOD mRNA among treatments, except the down-regulation in the high-FM treatment (with AM0 and OM). A three-way interactive effect (P = 0.030) of dietary FM, AM, and MM was found on Cu/Zn-SOD mRNA level. The gene expression of hepatic GPx-4 was significantly affected by dietary AM only, with its lowest level in LMB fed AM100 diets. The antioxidants mRNA levels showed similar trends to the activities of antioxidant enzymes in this study. The regulation of hepatic antioxidant genes expression in LMB fed various dietary treatments in this study could be related to the nuclear factor erythroid 2-related factor 2 (Nrf2) signaling pathway that play a vital role in the regulation of antioxidant enzymes gene transcriptions in fish^9^. Furthermore, though we did not find significant effect of MM source as a single factor (organic and inorganic) on the antioxidant defense of LMB, the interactive effects of FM, AM and MM on the Cu/Zn-SOD mRNA level in this study suggest that the MM (individually or in combination) could be involved in the antioxidant defense of LMB fed low-FM diets. Other studies reported that dietary MM supplemented in OM forms were more effective than IM forms in the antioxidant defense of other fish species^5,47^. The possible reason for the different findings of these studies could be due to differences in experimental design, fish species, minerals source and other factors.

TNF-α is pro-inflammatory cytokines, whereas TGF-β1 is anti-inflammatory^9,48^. In this study, interactive effects of dietary FM and AM were found on the relative gene expression of hepatic TNF-α and TGF-β1. Concurrently, an inverse trend was observed between the relative gene expressions of TNF-α and TGF-β1, with dietary AM (especially at 50% FO replacement) down-regulating TNF-α mRNA and up-regulating TGF-β1 in fish fed low-FM diets; although not significant for the former. The regulation of these genes by dietary FM and AM could be mediated by the signaling molecules of nuclear transcription factor-κB (NF-κB)^49^ and target of rapamycin (TOR)^8^, although this needs further elucidation.

FASN is a key enzyme for the *de novo* synthesis of fatty acids^50^. CYP7A1 is involved in cholesterol metabolism or elimination^51^ through catalyzing the first and rate-limiting step in the classic pathway of bile acid synthesis^52^. In this study, AM up-regulated the mRNA levels of hepatic FASN in LMB fed low-FM diets. Interactive effects of dietary FM and AM were also detected on the relative gene expression of hepatic FASN, while the expression of CYP7A1 mRNA was regulated by dietary AM only, with its augmented levels in LMB fed AM100 diets. A previous study reported that the mechanism of bile acids regulating the CYP7A1 expression is mainly through the farnesoid X receptor signaling (FXR)/ small heterodimer partner (SHP) pathway (FXR/SHP pathway) in the liver^53^. Our study proved that dietary AM could play an important role in lipid metabolism and cholesterol elimination in the liver of LMB through *de novo* synthesis of fatty acids and bile acids. Nevertheless, to our knowledge, this is the first report regarding the effect of dietary AM on FASN and CYP7A1 mRNA levels in the hepatic cells of fish, and the specific mechanisms behind the regulation of *de novo* synthesis of fatty acids and bile acids (FASN and CYP7A1 augmentation), cholesterol elimination, and promotion of dietary lipids metabolism in LMB needs further investigation.

Furthermore, in previous studies, the relative expression levels of the GH-IGF system were effectively used in the evaluation of organ growth and, ultimately, fish growth in response to different dietary treatments and alternative feed ingredients^33,54,55^. In this study, the relative expression of hepatic IGF-I was regulated by the dietary treatments. Specifically, IGF-I was significantly augmented in LMB fed the high-FM diet (with AM0 and OM) but was not different from those fed the low FM and AM50 diets. Interestingly, interactive effects of FM, AM, and MM were found on the relative expression of IGF-I mRNA. Thus, the relative expression of this signaling molecule could supply some important insights in understanding the underlying mechanisms for liver growth and, ultimately, the growth of LMB fed low FM diets supplemented with AM and MM.

## Conclusion

The results of this study indicated that dietary AM could completely replace FO (even in low-FM diets) and improve/ maintain the immune, antioxidant, anti-inflammatory, and lipid metabolism capacity of LMB. Similarly, the MM (individually or in combination) could be involved in the antioxidant defense of LMB fed low-FM diets. Indeed, dietary AM can be used as a good alternative to FO without detrimental impacts on the health of LMB, especially when it is supplemented with MM. Moreover, the relative expressions of hepatic Cu/Zn-SOD, GPx-4, TNF-α, TGF-β1, FASN, CYP7A1, and IGF-I mRNA were regulated by the dietary replacements, with interactive effects on the mRNA levels of Cu/Zn-SOD, TNF-α, TGF-β1, FASN, and IGF-I. These signaling molecules could aid in the understanding of the mechanisms behind antioxidant, inflammatory, and lipid metabolism status and organ growth of LMB in responses to dietary FM, AM, and MM. Furthermore, despite no significant effects of MM source as a single factor were identified for any of the response parameters reported herein, we believe that the observed interactive effects among FM, AM, and MM found contribute relevant information to the literature. Overall, our findings provide new insights for future studies on fish nutrition, particularly those geared towards the optimization of nutritionally balanced, cost-effective and environmentally friendly commercial feeds for LMB and other farmed species.
